# Convolutional Neural Networks for the Automatic Identification of Plant Diseases

**DOI:** 10.3389/fpls.2019.00941

**Published:** 2019-07-23

**Authors:** Justine Boulent, Samuel Foucher, Jérôme Théau, Pierre-Luc St-Charles

**Affiliations:** ^1^Department of Applied Geomatics, Université de Sherbrooke, Sherbrooke, QC, Canada; ^2^Vision and Imagery Team, Computer Research Institute of Montréal, Montréal, QC, Canada; ^3^Quebec Centre for Biodiversity Science (QCBS), Montreal, QC, Canada

**Keywords:** convolutional neural networks, deep learning, precision agriculture, Review (article), plant diseases detection

## Abstract

Deep learning techniques, and in particular Convolutional Neural Networks (CNNs), have led to significant progress in image processing. Since 2016, many applications for the automatic identification of crop diseases have been developed. These applications could serve as a basis for the development of expertise assistance or automatic screening tools. Such tools could contribute to more sustainable agricultural practices and greater food production security. To assess the potential of these networks for such applications, we survey 19 studies that relied on CNNs to automatically identify crop diseases. We describe their profiles, their main implementation aspects and their performance. Our survey allows us to identify the major issues and shortcomings of works in this research area. We also provide guidelines to improve the use of CNNs in operational contexts as well as some directions for future research.

## 1. Introduction

Plant health and food safety are closely linked. The Food and Agriculture Organization of the United Nations (FAO) estimates that pests and diseases lead to the loss of 20–40% of global food production, constituting a threat to food security (Food and Agriculture Organization of the United Nation, International Plant Protection Convention, 2017). Using pesticides is a way of protecting crops from these infestations and thus preserve yields. Their use has been one of the factors behind the increase in food production since the 1950s, enabling it to meet the needs of a growing population (Cooper and Dobson, 2007). However, the use of such substances is not environmentally harmless. Applying these substances negatively impacts biodiversity, including insect, bird, and fish populations, as well as soil, air, and water quality (Risebrough, 1986; Gill and Garg, 2014; Goulson, 2014; Sanchez-Bayo and Goka, 2014; Knillmann and Liess, 2019). Their use also constitutes a risk to human health, with both acute and chronic effects (Weisenburger, 1993; Bassil et al., 2007; Kim et al., 2016). However, the quantity of pesticides used is increasing worldwide, with +78% of tons of active ingredients used between 1990 and 2016 (Food and Agriculture Organization of the United Nation, 2018).

Knowledge of a fields' phytosanitary conditions is a decisive factor in limiting the use of pesticides while protecting harvests. Indeed, it enables farmers to carry out proper practices in the right place and at the right time. However, assessing the healthiness of fields is not simple, and it requires a high level of expertise. Indeed, a disease can be expressed differently from one plant species to another, or even from one variety to another. A given symptom may be the result of different problems, and these problems may also combine on the same plant. Even nutritional deficiencies and pests can produce symptoms similar to those of some diseases (Barbedo, 2016). Assessing the healthiness of plots is also time consuming. Checking the condition of each plant several times in a season is not practical on large farms. The difficulty of accessing some crops can also complicate prospection. The automatic identification of diseases by imagery has the potential to solve all these issues by using automatic prospection or expert assistance tools.

Determining the healthiness of a plant through an image is, however, a very difficult task. Indeed, crops are rich and complex environments. Their evolution is constant, with leaves, flowers, and fruits changing throughout the season. Their appearance also slightly changes during the day, as the amount and angle of incident solar radiation impacts their spectral response. Several techniques have been used to develop identification methods for crop diseases, whether under controlled or real conditions. These techniques were based in particular on the analysis of visible and near-infrared reflectance, on the development of specific vegetation indexes or even by pattern analysis. For more information on these techniques (see Sankaran et al., 2010; Mahlein et al., 2012; Martinelli et al., 2015; Barbedo, 2016). Those studies also identify several issues that block the effective use of these techniques for the automatic identification of diseases. Some of these issues are operational in nature and relate to image acquisition, weather constraints, deployment costs, availability, processing speed, and real-time diagnostic capabilities. Analyzing images from fields adds other issues, such as the ability to process complex elements like foliage or non-uniform backgrounds. Other bottlenecks are linked to the complexity of phytosanitary problems such as symptom variation over time and between varieties, or to the possibility of multiple disorders appearing simultaneously. Techniques capable of overcoming these challenges are needed to produce operational automatic diseases identification solutions. Since 2012, Deep Neural Networks (DNNs) and in particular Convolutional Neural Networks (CNNs) have been very successful in various computer vision tasks, such as object detection and recognition, classification, and biometry. The convolution layers of a CNN can be seen as matching filters that are derived directly from the data. CNNs thus produce a hierarchy of visual representations that are optimized for a specific task. As a result of CNN training, a model is obtained—a set of weights and biases—which then responds to the specific task it was designed for. One of the major strengths of CNNs is their capacity of generalization—that is, the ability to process data never observed before. This enables a certain robustness to background heterogeneity, to image acquisition conditions and to intra-class variability. However, learning those visual representations involves large-scale training data. Given their large number of parameters, one common problem of DNNs is their tendency to overfit the training data, which means that they become unable to generalize (Alom et al., 2018). The choice of the architecture adapted to a specific problem and the interpretability of the training results (black box effect) are other challenges surrounding CNNs. For more details about CNNs please refer to the following references: LeCun et al. (2015) and Goodfellow et al. (2016).

The purpose of this article is to synthesize the studies that have used CNNs to automatically identify crop diseases from images and to assess their potential for operational tools. This paper is organized as follows. The research method used to form our analysis corpus is detailed in section 2. After describing selected studies' profiles, section 3 presents the main aspects of the implementation of CNN-based methods, as well as their performance. In section 4, we focus on techniques that can help us better understand the trained models in order to avoid the black box effect and to ensure the reliability of the obtained results. Finally, in section 5, we highlight good practices based on both our experience with CNNs and on conclusions from other application areas. Future research directions are also proposed.

## 2. Research Methodology

The literature search was conducted through SCOPUS for works that matched keywords such as “deep learning,” “deep neural network,” or “convolutional neural network,” along with keywords regarding “diseases,” and “plants” or “crops.” The references of the selected articles were also checked. Only English-language articles published in established peer-reviewed journals through December 2018 were selected. The search was limited to studies using RGB images and supervised learning. Nineteen articles met our criteria. Synthesis tables describing these articles are presented in the Appendix.

## 3. Deep Learning Applied to Diseases Identification

### 3.1. Selected Studies' Profile

**Figure 1:**
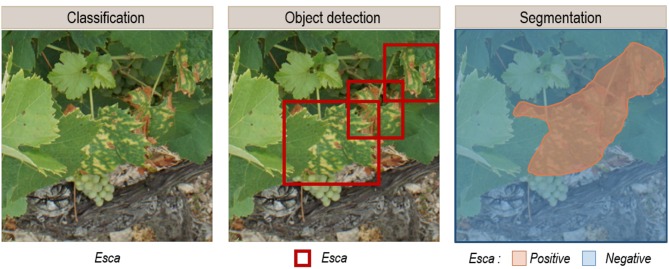
Expected output examples of **(A)** the classification, **(B)** the object detection, and **(C)** the segmentation of images containing esca disease symptoms.

In the selected corpus, there was a strong interest in market gardening, with tomatoes in 10 of the 19 selected articles. The issue of automatic crop disease identification can be addressed in a general or in a specialized approach. In the general approach (6/19), multi-crop and multi-disease models are trained, while the specialized approach focuses on one crop (13/19). The main similarity of these studies was their focus on analyzing a single organ: the leaves. Only two studies integrated other plant parts (Fuentes et al., 2017, 2018). The selected corpus mentions two application delivery approaches that motivate the development of automatic diseases identification solutions. The first approach is based on the use of mobile expertise tools that provide in-the-field identification capabilities. This approach relies on pictures taken with a regular hand-held camera and centered on leaves. The analysis tools are based on image classification, where a class or category label is assigned to each analyzed image (Figure 1A). Ramcharan et al. (2017) and Picon et al. (2018) implemented mobile applications to use their models in the field. The second application delivery approach is based on automatic phytosanitary monitoring via autonomous vehicles. This is not presented in concrete terms but rather as a development perspective. In addition, to achieve a working model under field conditions, the data used for training must reflect the complexity of the studied environment. Diseases, plants, and crops in general are dynamic objects and environments whose appearance can change. Several phenological or symptom development stages, backgrounds, light conditions, and even several acquisition scales (one organ, one plant, several plants) must be integrated. It is also necessary to not only identify diseases but also to locate them. Classification therefore does not produce adequate output when used without a localization solution. To obtain their location, two approaches can be used: object detection, which provides identification and location as a bounding box (Figure 1B), or segmentation, which provides identification for each pixel in the given image (Figure 1C). In the corpus, 16 studies perform classification and 3 use object detection.

### 3.2. Dataset Design

#### 3.2.1. Data Origin and Characteristics

**Figure 2:**
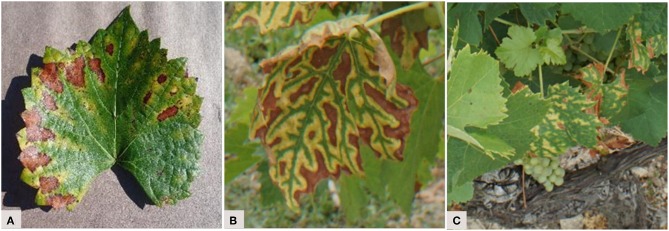
Typology of image complexity found in the datasets. Esca grape disease on **(A)** an image captured under controlled condition (from the PlantVillage dataset), **(B)** an image captured under uncontrolled condition and with a focus on a particular organ, and **(C)** an image captured under uncontrolled conditions and without focus on a particular organ.

The quantity of information and the diversity within the images varies among the studies. Three types of datasets can be defined, depending on their level of complexity (Figure 2). The first type consists of images captured under controlled conditions. In this case, images show one leaf picked up in the field and placed on a uniform background, in an environment with controlled illumination (Figure 2A). This simplifies the image analysis process by removing any variability related to external conditions or plant morphology in order to focus on symptom expression. A total of 13 of the 19 studies used such images. The second and slightly more complex type of dataset consists of images captured under uncontrolled conditions, but focusing on a particular plant organ, generally a leaf. In this case, images have a complex background but the largest area is occupied by the object of interest (Figure 2B). Only 3 of the 19 studies used such images. Finally, the last type of dataset consists of images captured under uncontrolled conditions and without focusing on a particular plant organ. These images therefore reflect what an operator would see in the field, with all the complexity associated with foliage architecture (Figure 2C). This kind of dataset is the one best-suited to build an operational automatic phytosanitary monitoring tool. Only 3 of the 19 studies used such images.

A majority of the images used in these studies come from public datasets (11 out of 19 studies). The most widely used dataset is PlantVillage, a database initially described in Hughes and Salathé (2015) and now containing 87,848 photographs of leaves of healthy and infected plants (Ferentinos, 2018). A total of 25 species are represented through 58 classes, with 62.7% of the images taken under controlled conditions. Barbedo (2018b) used an open database containing 1,383 images of 12 plant species through 56 classes. Its acquisition conditions are mainly controlled. On the other hand, DeChant et al. (2017) used a dataset captured under uncontrolled conditions and with no focus on a particular plant organ. This dataset is highly specialized in the identification of Northern Leaf Blight (NLB) infected maize plants. A description of an extended version of this dataset can be found in Wiesner-Hanks et al. (2018). It consists of 18,222 annotated handheld, boom and drone images with 105,705 NLB lesions. The availability of such databases is significant, as it provides a high number of annotated images—a key factor of success in deep learning. These images can also be used for benchmarking, enabling a comparison of the accuracy of models created by different research groups. Collecting images in the field and gathering them into databases such as PlantVillage is an ideal solution to improve the community's research capabilities.

The images in private datasets (the type utilized by eight studies) were all handheld. A weakness of such datasets is that they sometimes lack important details, such as the capture conditions, acquisition date, varieties studied and the intensity of the analyzed symptoms. In some studies, too few or even no samples are illustrated, making it impossible to determine the application prospects of the trained models. Indeed, a model trained with a low-complexity image dataset (as defined earlier) will not be able to generalize to data from a more complex setting. This was underlined by Mohanty et al. (2016), whose accuracy went from 99.35% on a held-out test set to 31% on a test set with images taken under different conditions. The work of Ferentinos (2018) divided the PlantVillage dataset based on either laboratory or field conditions. Their model trained on laboratory images reached 33.27% accuracy when applied on field images, while their model trained on field images reached 65.69% accuracy when applied on laboratory images. As data is the key element of a successful CNN-based model, its characteristics and source(s) must be well described.

#### 3.2.2. Class Taxonomy Definition

Classes are defined by diseases and species (in the case of multi-species models). In Wang et al. (2017), classes reflect disease severity levels. Both the intensity and the stages of infection can lead to a high degree of variability in symptoms. This variability can be expressed through separate classes (as did Wang et al., 2017) or integrated in global classes. However, since it is difficult to obtain enough images for all expressions, especially over a single growing season, Barbedo (2018a) recommends the continuous addition of new images to the training dataset. The number of classes vary greatly from one study to another—from 2 (DeChant et al., 2017) to 58 (Ferentinos, 2018). Four studies also dedicated a class to the background. Adding such a class is meaningful for a real world application, where the background is not uniform. To this end, Sladojevic et al. (2016) and Brahimi et al. (2018) used the publicly available Stanford Background dataset (Gould et al., 2009). On the other hand, Fuentes et al. (2017) extracted patches of healthy plants and background from their images and put them together in a transversal class of negatives. They relied on hard negatives to form this class. Hard negatives are false positives obtained from previous evaluations that are integrated into the training set for the negative class for a new training. This practice aims to reduce the number of false positives by confronting the network to situations that it has previously failed to manage properly. The negative class is quite complex to construct, as it must integrate all the diversity of the real world without having an excessively higher number of images than the positive classes. Using hard negatives can help target the most relevant negative cases. Even if imbalance is a reflection of the field reality where healthy plants are in the majority, some classes should not have a much higher number of examples than others. The class imbalance problem affects both the convergence of the model and its ability to generalize (Buda et al., 2018). Different strategies can be chosen either at a data level or at an algorithm level to minimize the detrimental effect of imbalance (Krawczyk, 2016). Wang et al. (2017) chose undersampling. They divided their healthy leaves' class into 12 clusters of 110 images for training and 27 for testing, thereby providing classes of the same size (between 102 and 144 for training and between 23 and 36 for testing). Having a taxonomy that only includes individual diseases is a simplification of reality. Very often, diseases, nutritional disorders, and/or pests can be present at the same time, combining their symptoms. Creating a class for each phytosanitary problem combination does not seem to be a suitable solution since the number of possible classes would increase considerably (Barbedo, 2018a). It would be unfeasible to collect enough images for each class. To solve this problem, Barbedo (2018a) proposes to consider lesions individually and process only areas of symptomatic interest identified by the user. Another possibility is to train binary models (target disease versus the rest) and thus give primacy of detection to the disease of interest, even if it does not express “pure” symptoms. In any case, the greatest challenge is to cover enough symptom expressions so that the model can be applied in real world conditions.

#### 3.2.3. Data Annotation

The association of a label to all or part of an image—is a laborious but unavoidable step in supervised learning. Repetitive and time-consuming, it must be carried out by an expert in identifying crop diseases, which makes this task difficult to delegate. The annotation method depends on the general approach chosen for image analysis. For classification, it consists of associating a label to each image, either by integrating it in the metadata or by organizing the images, e.g., into folders corresponding to the different classes. For object detection, the coordinates of the target within the image must be entered. This is done by delineating regions of interest that are often rectangular but may also correspond more precisely to the object in question.

**Figure 3:**
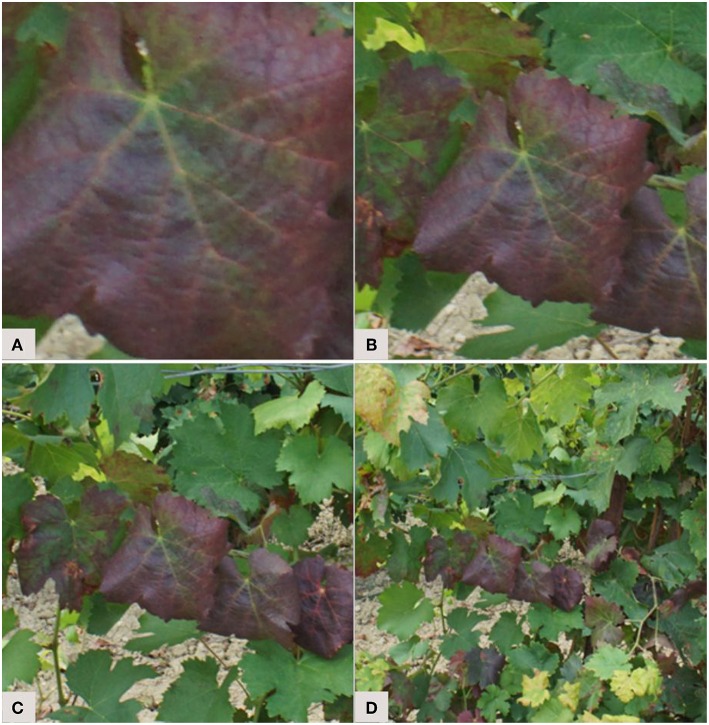
Selecting an analysis scale: from a scale close up on the main symptoms **(A)** to a scale providing more contextual features **(D)**. Example of a vine branch affected by *flavescence dorée*.

For the identification of diseases, the annotation step raises the question of the analysis scale and of the importance given to the context. Indeed, what is the best piece of information to send to the network? The lesion, the leaf, or the whole plant? Each of these levels are valid and provide complementary features. With a close symptom view, textural elements stand out (Figure 3A). A complete view of the leaf reveals symptom patterns (Figure 3B). Finally, a view of the whole plant provides a spatial perspective of the symptoms. For example, some problems occur preferentially on young leaves or infect a whole branch (Figures 3C,D). Some authors compared those scales and assessed their impact on the final results. Ramcharan et al. (2017) formed two datasets: an “original cassava dataset” with entire cassava leaves, and a “leaflet cassava dataset” where the leaflets were manually cropped. The accuracies obtained were slightly higher at the leaflet scale for three of the five studied diseases. Leaf cropping thus had no significant impact, despite the fact that the “dataset leaflet” was seven times wider than the original. Picon et al. (2018) addressed the early identification of wheat diseases. Three ways of extracting the tiles sent to the network were tested. The first downsampled the image to match the size required by the network. The second approach was to crop a rectangle containing only the leaf. The third approach, called “superpixel based tile extraction,” was based on the segmentation of the image into homogeneous zones. On the test set, the balanced accuracy value was higher at the superpixel scale for one of the two diseases tested. There is therefore no ideal amount of context to give, as it varies from one object of study to another. It is however interesting to note the potential of multi-scale approaches that benefit from information captured at different scales. Note also that a random scaling factor can be added during data augmentation to help models generalize to scale variations (see section 3.3.3).

#### 3.2.4. Dataset Division

When using deep neural networks, three separate datasets are required to develop a model. The first set, the training set, is the collection of images to be used by the network to automatically learn its hidden parameters, such as weights and biases. The second set, the validation set, is used to manually adjust hyperparameters, which are essentially the settings that cannot be automatically learned during training. These include among others the learning rate, the batch size and the network architecture. For more information about hyperparameters (see Goodfellow et al., 2016). The values of these hyperparameters are often set empirically, as they are linked to the problem, the dataset, and the model architecture. Therefore, there are no good predefined values, as they must be tuned based on the performance (in terms of accuracy) obtained on the validation set. This means that information about the validation data indirectly leaks into the model, resulting in an artificial ability to perform well on these images (Chollet, 2017). For that reason, the validation images should only be used to tune the hyperparameters; the final evaluation of the model's performance is done using the test set, discussed in the next paragraph. The model being trained can be evaluated on the validation set at the end of each epoch, allowing the training process to be monitored and to detect overfitting. The training and validation sets come from the same data source that is subdivided. Most of the images go for training (between 70 and 85% depending of the size of the dataset). Mohanty et al. (2016) and Zhang S. et al. (2018) tried five different separation ratios and both concluded that using 80% for training and 20% for validation was ideal for their data. Another way to divide the images into training and validation sets is cross validation. The dataset is divided into several subsets that are randomly used for training or validation. Cross validation is useful when the dataset is small as it avoids any result bias caused by an arbitrary predetermined and fixed data separation. It is used in 5 of the 19 selected studies. Once the hyperparameters have been defined, further training can be done by gathering the training and validation sets to benefit from a greater number of images.

The third dataset that is needed is the test set. It is used when the training phase has been completed, with the objective of evaluating the model's final generalization ability. The accuracy on the test set is thus the most important metric to compute, as it provides an overview of the model's performance beyond the hyperparameter exploration process. The test set must be independent from the training and validation sets, so it cannot be obtained from a simple subdivision. However, 6 of the studies we analyzed (31.5%) formed their test set this way. Worse yet, 11 of the studies (58%) did not even have a test set. Only 2 studies (10.5%) performed evaluation on an explicitly different test set. Having those three datasets is essential, since the observed data variability in the agricultural setting is quite important. Therefore, there must be a way to ensure that the generated models can operate under different conditions and in different fields. Using data acquired on a different plot for the test set can be a good and simple way to achieve data independence. Even though obtaining data in an agricultural setting can be complex, it should not lead to overlooking this critical aspect.

#### 3.2.5. Data Pre-processing

Before sending images to the network, two pre-processing steps are often necessary. First, the images must typically be resized to match the size of the input layer of the CNN. The sizes are quite standard from one network to another, with for example 227 × 227 for AlexNet, 224 × 224 for DenseNet, ResNet, and VGG, and 299 × 299 for Inception. Secondly, the images must be normalized to help the model to converge more quickly as well as to better generalize on unseen data (Chollet, 2017). Other pre-processing operations have been proposed. Mohanty et al. (2016) and Oppenheim and Shani (2017) transformed their images to grayscale. Mohanty et al. (2016) compared accuracies obtained on grayscale with those from color images. The performance was slightly higher on the color models, with the f1-score improving from 1.34 to 3.33% (for details on the f1-score, see Powers, 2011). Even if using color images helps the identification process, as the performance decreases only slightly during the grayscale transformation, this highlights that the network relies mainly on other features to identify diseases. In the same study, the authors also evaluated the impact of background suppression. In fact, background management is one of the challenging elements in the implementation of automatic methods for identifying phytosanitary problems in imagery. With conventional image processing methods, leaf segmentation is a preliminary step to the analysis (Barbedo, 2016). The performance obtained by Mohanty et al. (2016) is marginally better with the background, improving the f1-score by slightly <1%. Since background segmentation is not an option on images taken in the field, and since it is the strength of the CNNs to manage complex backgrounds, background suppression is unnecessary.

### 3.3. Training Phase

During the training phase, the model's internal weights are automatically updated over several iterations. External factors such as the training strategy, architecture, regularization techniques, or the value of the hyperparameters influence this training process.

Comparing studies and their results to extract insights on how to define the training phase is complicated because they do not use the same data and they do not provide all the parameters required to reproduce their experiments. It is also difficult to appreciate the significance of the conclusions made in these studies because their experiments are not performed multiple times to evaluate the impact of random initializations and training sample ordering. Nevertheless, we decided to present some comparisons of training and architectural strategies while keeping in mind that some of their results are potentially biased.

#### 3.3.1. Training Strategies

There are two ways to train a CNN: from scratch or with transfer learning. Transfer learning is when a network that is pre-trained on a large set of images (for example ImageNet, and its 1.2 million images in 1,000 classes) is used and adapted to another task. This kind of learning is enabled by the fact that the first layers of CNNs learn generic low-level features that are not class specific (Zeiler and Fergus, 2014). In practice, this adaptation is done using the network weights from previous training. Using transfer learning allows us to use CNNs even when the amount of training data is limited, which is often the case in the context of crop diseases identification. This technique helps to achieve greater generalizability, as the network had previously learned to deal with millions of examples. It is also a way to save in terms of computing time and capacity. There are two ways to perform transfer learning: by feature extraction and fine-tuning. Feature extraction consists in keeping the weights of a pre-trained model intact and using the embeddings it produces to train a new classifier on the target dataset. Fine-tuning consists in using the weights of a pre-trained model to initialize the model and then training all or part of these weights on the target dataset (Chollet, 2017). Choosing one technique or the other depends in particular on the proximity between both the source and target datasets (in case they are very close, feature extraction may be sufficient) but also on the size of the target dataset. Training a large number of layers with a small dataset may increase the risk of overfitting. Training from scratch is when the network weights are not inherited from a previous model but are instead randomly initialized. It requires a larger training set, and the overfitting risk is higher since the network has no experience from previous training sessions and so must rely on the input data to define all its weights. However, this approach allows us to define a problem-specific network architecture that can improve the performance. These problem-specific architectures can be developed, for example, to handle more than three color channels, multi-scale dimensions, or to integrate multiple models trained differently (with dissimilar hyperparameters or datasets). In our corpus, 15 studies (79%) used transfer learning and 7 studies (37%) trained a model from scratch.

**Figure 4:**
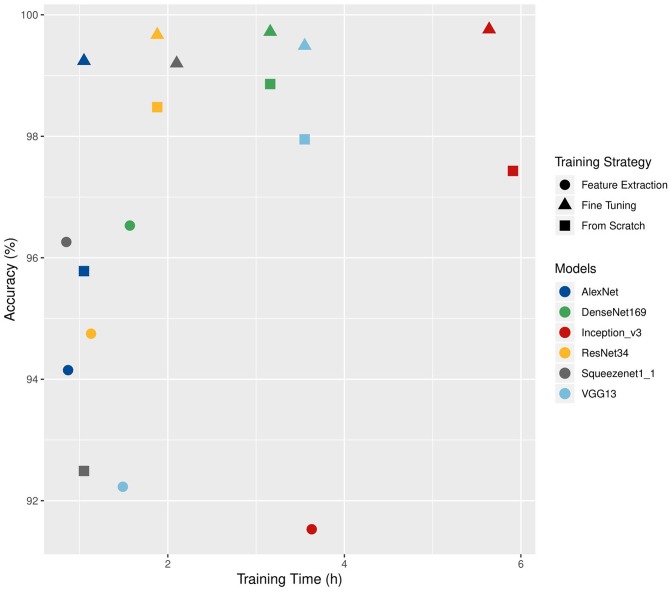
Comparison of the training time (h) and accuracy values (%) obtained on a validation set according to different architectures and training strategies. Adapted from Brahimi et al. (2018).

Choosing a training strategy depends on both technical (amount of available images, computing capacity) and thematic (availability of a suitable architecture or of pre-trained weights compatible with the data used) considerations. Brahimi et al. (2018) compared three training strategies on six CNN architectures (AlexNet, DenseNet-169, Inception v3, ResNet-34, SqueezeNet-1.1, and VGG13). They used the PlantVillage dataset augmented with a background class. Two strategies used transfer learning: feature extraction and complete fine-tuning. In the third strategy, the network was trained from scratch. The accuracies obtained on the validation set and the training time are shown in Figure 4. For the six architectures, fine-tuning gave the highest precision (from 99.2% for SqueezeNet to 99.5% for VGG13). The times required for fine-tuning and for training from scratch are close (from 1.05 to 5.64 h for fine-tuning and from 1.05 to 5.91 h when trained from scratch). The feature extraction approach had the lowest training times (from 0.85 to 3.63 h).

Overall, training from scratch and transfer-learning should not be seen as completely mutually exclusive strategies. In case of a new architecture where no prior weights are available, pre-training may still provide some benefits. The idea is to start by training the model from scratch on a large dataset (like ImageNet, but also PlantVillage or another plant database) and then carry out fine-tuning on the specific data gathered for the study (Cruz et al., 2017; Picon et al., 2018).

#### 3.3.2. Architectures

CNNs are based on three main components: convolutional layers, pooling layers and activation functions, commonly Rectified Linear Units (ReLUs). The number of layers used, their arrangement and the introduction of other processing units vary from one architecture to another, determining their specificity. In 17 of the 19 selected studies, state-of-the-art architectures were used. Architectures proposed alongside the first popular CNNs (e.g., LeNet, AlexNet, and CaffeNet) are used in 11 studies. One study used SqueezeNet, known to achieve similar performance as AlexNet on ImageNet with 50 times fewer parameters. The acclaimed ResNet, VGG, and Inception architectures are used in 15 studies. Newer architectures such as DenseNet or ResNetXt are used in three studies. For more information about architectures, see the work of Khan et al. (2019).

**Figure 5:**
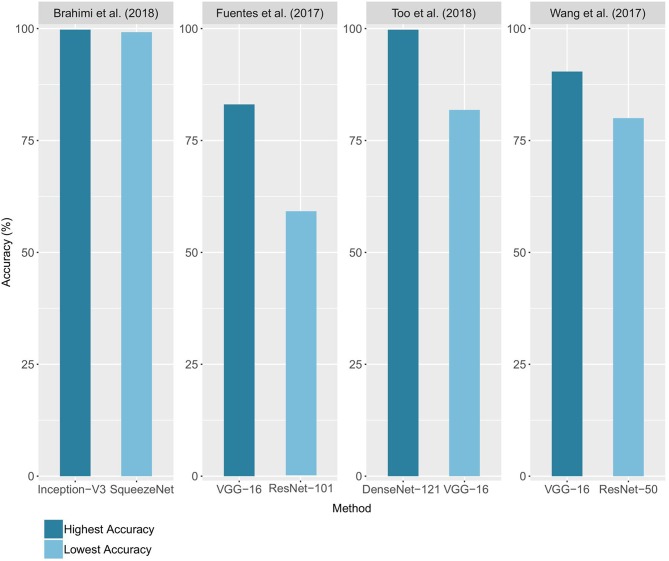
Comparison of the accuracies obtained using different architectures. For each study, the lowest and highest performance achieved on the validation set (or test set when available) is reported.

In 12 of the 19 studies, different architectures were compared, with the highest and lowest accuracy for 4 of these studies reported (Figure 5). The accuracies range from 59% with a ResNet-101 (Fuentes et al., 2017) to 99.75% with a DenseNet-121 architecture (Too et al., 2018). Network complexity and depth do not necessarily lead to higher accuracy, as shown by the superiority of the VGG-16 results over those of ResNet-101 for Fuentes et al. (2017) and the closeness of the results of Inception-V3 and SqueezeNet (Brahimi et al., 2018). The performance reported for a unique architecture may also vary from one study to another, as in the case of VGG-16, which ranked as the best architecture for Wang et al. (2017) and Fuentes et al. (2018), but as the worst for Too et al. (2018). Since architecture implementations are now widely distributed through standard libraries such as PyTorch and Tensorflow/Keras, we advise trying several architectures to find the ones that bring the best results on a studied case. This choice will depend on the nature of the data, its quantity and the time and resources available for training. Choosing an architecture and defining the optimal values of the other hyperparameters can seem like a hazardous trial-and-error process. However, there are methods to guide this process, whether through manual hyperparameters tuning, random or grid research (Bengio, 2012; Goodfellow et al., 2016; Smith, 2018).

Custom architectures were used in seven studies. In some cases, this involved adapting a reference architecture so that it is more efficient in handling the study data. To limit the risk of overfitting due to their small dataset, Oppenheim and Shani (2017) relied on drop-out, a regularization technique based on the random disconnection of links between model layers. In other cases, architectures were more customized. Cruz et al. (2017) implemented what they called an “Abstraction Level Fusion.” They injected manually generated features into the fully-connected layers of the network in order to guide its training and possibly accelerate it. Zhang S. et al. (2018) presented a three-channel convolutional neural network with the goal of improving the use of color information. DeChant et al. (2017) developed a three-stage process based on the training of several CNNs to compute heat maps which are used to determine if the analyzed image contain diseased lesions. Fuentes et al. (2018) implemented a framework based on CNN filter banks to minimize the number of false positives. Customized architectures can be useful to obtain a model more adapted to a specific study case. However, a customized architecture must be compared to a recent state-of-the-art architecture in order to measure its unique contribution.

#### 3.3.3. Regularization Techniques

The main challenge in machine learning is to obtain a trained model that is able to analyze new and unseen data. This aspect is far from being guaranteed by high training accuracy. Indeed, the main pitfall in deep learning is overfitting. This occurs when the number of input samples is too small compared to the learning capacity of the network. Overfitting does not allow to learn the general characteristics of the classes and instead captures the noise of the training set (Srivastava et al., 2014). This leads to a model with high accuracy during training but that is unable to generalize (i.e., it does not achieve high test accuracy). In the corpus, trained models are not systematically tested on independent data (only two studies have an explicitly independent test set). It is therefore, not possible to determine whether the models were overfitting. However, several of the selected studies presented a low minimum number of samples per class (before augmentation): equal to or less than 55 for 2 studies, and between 55 and 200 for 8 studies. In those cases, the number of images seems too small to train a model in a robust way—especially considering the diversity in the plant world.

To improve model generalization, the first and obvious step is to gather more data. However, obtaining many images for a given class can be complicated in an agricultural context, especially when it comes to diseases. In machine learning, techniques have been developed to improve the performance of the test set, even if it means reducing the performance of the training set. These techniques are called regularization techniques (Goodfellow et al., 2016). One of these is data augmentation, which consists of the transformation of the geometry or intensity of the original images to make them seem like new images. The operations are often simple: rotation, mirroring, random cropping, zooming, or even adding noise, changing contrast, and brightness values as well as simulating new backgrounds. The size of the dataset and its diversity are therefore artificially increased. The augmentation operations can be performed in different ways, using one or several (possibly randomly chosen) transformations per image. The transformations can also be applied before the start of the whole training process, or “online” when each image batch is uploaded. An incorrect practice noted in some studies must be highlighted: the augmentation was sometimes carried out before the separation of the images into training and validation sets. It is important to carry out these augmentation operations once the sets have been defined to ensure that an image and its duplicates are in the same set. Additional techniques applied to the model itself, such as drop-out (detailed in section 3.3.2), or during the training process, such as weight decay and early stopping, were found in the articles. The use of such techniques is recommended.

### 3.4. CNN Performance

#### 3.4.1. Comparison With Other Approaches

**Figure 6:**
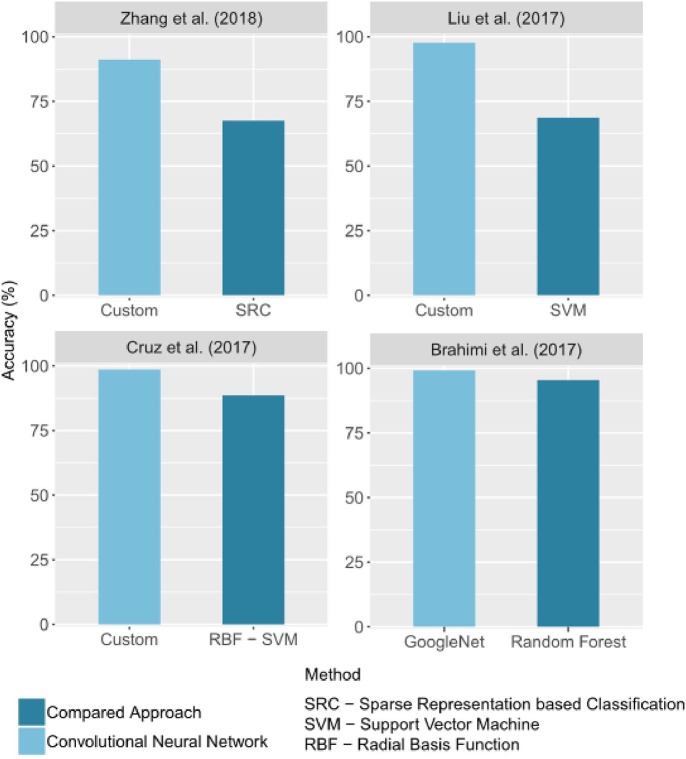
Comparison of accuracy values obtained by CNNs and by other image processing methods. Only the best CNN architecture and comparative approach for each study were reported. If given in the study, the precision on a test set was reported.

In image classification, CNNs outperform traditional image processing methods in several applications. This general trend is also observed in the automatic identification of crop diseases. Some of the selected studies compared the performance obtained with CNNs to that of other methods. In all of these studies, the CNN results are better than the others. Figure 6 groups the best results obtained for a CNN and for an alternative method in studies that made a comparison. The difference of accuracy ranged from 3% (Brahimi et al., 2017) to 28.89% (Liu B. et al., 2017).

#### 3.4.2. Generalization Performance

To assess the generalizability of a model, it must be evaluated on a dataset of images never seen before, which is the test set mentioned in section 3.2.4. Only 2 of the 19 studies selected relied on an explicitly independent dataset to conduct this evaluation. Mohanty et al. (2016) trained a model to identify 14 crop species and 26 diseases. They used the PlantVillage database. After an accuracy of 99.35% on a held-out test set, they performed an evaluation on two new datasets of 121 and 119 images, respectively, downloaded from the Internet. On these datasets, they got an accuracy of 31.40% for the first and 31.69% for the second. Even if this accuracy is higher than random guessing, it is insufficient for practical use. It can be assumed that the training led to overfitting and/or that the input dataset lacked diversity compared to the test set. Picon et al. (2018) aimed to identify three wheat diseases. Using a mobile application, technicians tested the model for two of the three diseases studied: Septoria (77 images) and Rust (54 images), to which they added 27 images of healthy plants. A balanced accuracy of 98% was obtained for Septoria, and of 96% for Rust. No drop in performance was observed. This solid performance can be explained by the fact that the test images were acquired at the end of the season—where the symptoms are most visible—but also thanks to good training practices. Indeed, acquiring more than 1000 samples per class, in several plots in Spain and Germany, over three seasons (2014, 2015, and 2016) and in real environments resulted in enough diversity for the model to work well in operational conditions. These two studies emphasize once again the importance of adopting good training practices and defining an independent test set to properly evaluate the performance of CNNs and their ability to be used operationally.

## 4. Understanding the Trained Models

One of the downsides associated with deep learning methods is the difficulty in understanding what the model has learned—the famous black box effect. Still, it is possible to understand the model's inner mechanism or at least get a glimpse of it through several techniques. By better understanding the trained models, we can not only ensure the relevance of the results generated, but also improve their quality.

### 4.1. Standard Techniques

Easy-to-implement methods that can assess the quality of prediction and gain an insight into how the training progressed have been found in the studied corpus. First, additional metrics to the overall accuracy may be calculated, such as the level of confidence in its prediction and the sensitivity and specificity of the model. By using confusion matrices, inter-class confusion can be assessed. The analysis of incorrect predictions is used to find problematic situations that the model cannot handle. Thus, DeChant et al. (2017) observed that illumination variations, background leaves, dead ground vegetation, senescent leaves at the bottom of canopies and insects were badly handled by their model. Fuentes et al. (2017) observed confusion between classes with high pattern variations. Ferentinos (2018) pointed out that their model struggles to manage shadow effects, the presence of non-plant objects, and the case where the analyzed leaf occupies a small and non-central part of the image. They may also have found images that were not properly annotated. While these methods lead to a better appreciation of models results and provide clues for improvement, they do not ensure that the training is properly carried out and that the results are due to relevant features. For instance, in our case, these techniques cannot confirm that only plant lesions are used to identify the target diseases instead of other unrelated characteristics from the photos.

### 4.2. Visualization Techniques

**Figure 7:**
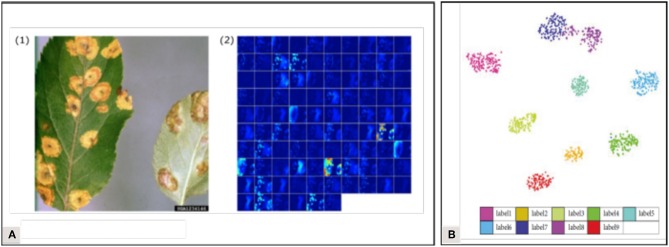
Visualization examples found in the corpus **(A)** Activations in the first convolution layer visualization (Mohanty et al., 2016). **(B)** T-distributed Stochastic Neighbor Embedding on the final fully connected layer (Zhang K. et al., 2018).

To improve the transparency of the learning process, several visualization methods have been developed, allowing us to picture what is happening in the network. Atabay (2017) used the occlusion technique which consists in sliding an occlusion window on the image to study the variation in the probability belonging to the right class. They pointed out that the class was sometimes assigned because of pixels belonging to the background—indicating that the features learned were not just those linked to the symptoms. Brahimi et al. (2018) also used the occlusion technique but underlined that it was computationally expensive and time-consuming. They computed saliency maps based on gradient values to estimate the pixels' importance in the node corresponding to the ground truth. They computed this in two ways: with and without guided backpropagation, where only the positive gradients are propagated through the activation functions, which helps to obtain more precise visualizations. Mohanty et al. (2016) visualized the top activated feature maps at the output of early convolution layers (Figure 7A). Zhang K. et al. (2018) used t-distributed Stochastic Neighbor Embedding (t-SNE) to visualize the features of their final fully connected layer and to evaluate the distance between their classes (Figure 7B). The insight brought by all these visualization methods can help us understand the behavior of trained models while suggesting new improvements. Their implementation minimizes the black box effect, solidifying the reliability that can be attributed to the models, which is decisive for an application in real agricultural conditions. Implementing visualization solutions has also been recommended in the medical image analysis field, where understanding the prediction system is crucial to ensure correct diagnostics (Litjens et al., 2017). For more details on visualization techniques (see Zeiler and Fergus, 2014; Qin et al., 2018).

## 5. Discussion

CNNs provide unparalleled performance in tasks related to the classification and detection of crop diseases. They are able to manage complex issues in difficult imaging conditions. Their robustness may now allow them to emerge from the research environment and become part of operational tools. However, before tools for expertise assistance and automatic screening become a reality, a few steps still need to be tested and integrated. In this section, we first discuss the best practices to adopt all along the development chain so that trained models are able to handle the real-world complexities of agricultural and phytosanitary problems. We then identify the elements to be further addressed to make such tools fully operational, including possible research directions.

### 5.1. Adopting the Best Practices

#### 5.1.1. Targeted Image Acquisition

The robustness of a trained model is linked to the quality of its training dataset. Data diversity is one of the key elements to ensure model generalization. Indeed, as also highlighted in Barbedo (2018a), the training dataset has to reflect the reality of the operational environment, which is very challenging. Considering the target application before data acquisition can allow us to capture more appropriate images. For example, for an automatic screening tool, the entire plant must be included in the image, while for an expertise assistance tool, only the leaf or the fruit can be focused on. The operational scope must also be defined in advance. For example, in the case of a tool operating on several varieties or even species, it is necessary to include all the different expressions of symptoms. Carrying out the acquisitions at different hours of the day and under several meteorological conditions ensures the model can operate free from these constraints. Ideally, images should also be acquired on several farms in order to be confronted with a variety of maintenance conditions. Using more than one camera avoids dependency on a particular device. Following this step, the nature of the test set should be considered to ensure that its images will be independent from those used in training. For this purpose, a plot can be devoted to produce the test set. The plot conditions must match the target application scenario, for example by including diseases other than the one studied.

#### 5.1.2. Dataset Preparation

The architecture of a model is not the main factor that must be considered to obtain good accuracy. It is rather the quality of the training data as well as its preprocessing and augmentation that can provide the most significant accuracy improvements (Litjens et al., 2017). All steps related to the preparation of the data must therefore be carried out in a rigorous way. The annotation phase should begin by an explicit definition of the class taxonomy, particularly if the contamination intensity is annotated. This step ensures ensures the annotations reproducibility. Having more than one expert for annotation prevents the risk of dependence on the annotator. Besides, augmentation operations have proven to be effective against overfitting. Easy to set up, they are fully encouraged. It is however imperative to perform these transformations after the separation into training and validation subsets. Otherwise, an image and its transformation could end up in both training and validation sets, causing data leakage. Dealing with the class imbalance is also important for the convergence of the model and its generalizability (see section 3.2.2).

#### 5.1.3. Training and Evaluation Phases

If the time and computing resources allow, conducting several training sessions with the same hyperparameters can lead to improved accuracy, as random initializations can have an impact on the results. When comparing hyperparameters, it would also be advisable to consider fixing the random number generators to prevent them from biasing the comparison. Experimenting with more than one type of architecture can also have a positive effect. For equal accuracy, choosing the least complex architecture is more advantageous from an operational point of view. If applicable, transfer learning is recommended to improve computation time and generalizability. Once all the hyperparameters have been fixed, the model should be retrained by combining the images previously used for training and validation into a global training set. Indeed, as soon as all the hyperparameters have been defined, there is no longer any reason to keep the validation set. It is then worth using this global training set to try to improve the accuracy one last time (i.e., without follow-up adjustments to any hyperparameters). The retrained model can then be evaluated on the test set. The visualization step is also important, as it helps to better understand what is happening in the model and to ensure the robustness of the results. This methodology can also provide opportunities to improve performance.

#### 5.1.4. Sharing Reproducible Results

To ensure the results obtained and shared are useful to the scientific community, it is important to have a reproducible research perspective, which is sometimes still lacking in artificial intelligence (Hutson, 2018). When working with CNNs, this requires the sharing of not only all of the hyperparameters, but also data, code, and even trained models. This sharing is not always possible because of commercial considerations, but it does allow reproducible works to gain a greater scientific appreciation (Peng, 2011) and to contribute to the progress of the research field.

### 5.2. Outlook

Most applications in crop diseases identification will emerge in uncontrolled environments. Efforts will therefore have to be focused on forming datasets similar to what is found in the field and preferably taken with the acquisition tool of the target scenario application—whether it is a smartphone, a drone, a robot or a tractor. Once new data is acquired, it commonly involves annotation. This step is quite tedious but it is possible to simplify it with active learning. Active learning is an iterative procedure designed to find and annotate the most informative samples. The concept behind this methodology is that annotating good examples can lead to similar or even better accuracy than annotating all examples, and for a lower operative cost as well as requiring less time. A model must first be trained from a small subset of annotated examples. This model is then queried to find the most informative samples (Settles, 2009). Already used as a tool to build datasets in machine learning, this technique is now used with deep neural networks, including for the annotation of medical images (Gorriz et al., 2017; Otálora et al., 2017) or in remote-sensing (Liu P. et al., 2017). Another way to reduce the annotation effort would be to share the annotated data. By combining data annotated by different experts, this would also prevent bias related to individual annotators, and it would generally help improve the quality of annotations. Robust and complete datasets could be formed this way. Nevertheless, annotation in such specialized areas is sometimes complex and it can even be difficult for an expert to identify the symptoms of a plant disease. Rigorous annotation is therefore challenging and associated with uncertainty, leading to difficulties in the use of images grouped by social networks (Barbedo, 2018a). Having more benchmarck datasets would be very valuable for our field. This recommendation is shared in other areas where annotation requires significant expertise, such as image processing in biology and medicine (Ching et al., 2018). An alternative approach is to avoid the annotation process entirely by using unsupervised learning algorithms for anomaly detection. Already investigated in the medical field (Chalapathy and Chawla, 2019), this could be explored for plant analysis.

In a more thematic way, one other aspect to explore is the early detection of disease. As the first symptoms are more difficult to detect, the use of cameras capturing infrared reflectance would be very interesting. Going beyond the identification model to a decision model is also a perspective. A tool that can identify the problem and provide recommendations for solving it could be a real asset for more sustainable agriculture. The output of the identification model could be just one of the inputs of the decision model, together with auxiliary features such as weather forecasts, geographical characteristics, plot contamination history, or disease diffusion pattern.

Next, the classification goal found in most of the corpus' articles is suitable for expertise tools but not for automatic screening. Indeed, for this second application, a localization of symptoms is also necessary. In this case, image-space detection or segmentation approaches can be used. Deployment models and user interfaces will also have to be designed. If onboard tools are developed to provide real-time diagnostics, light CNN architectures will be required. The question of minimum spatial resolution will also need to be explored. Close collaboration with farmers could lead to creating solutions fully meeting their needs and financial capacities. User feedback will be decisive, as it will enrich the models with new samples, leading to more robust identification.

## 6. Conclusion

In this paper, we identified some of the major issues and shortcomings of works that used CNNs to automatically identify crop diseases. We also provided guidelines and procedures to follow in order to maximize the potential of CNNs deployed in real-world applications. Many already-published solutions based on CNNs are not currently operational for field use mostly due to a lack of conformity to several important concepts of machine learning. This lack of conformity may lead to poor generalization capabilities for unfamiliar data samples and/or imaging conditions, which lowers the practical use of the trained models. Nevertheless, the studied works show the potential of deep learning techniques for crop diseases identification. Their findings are definitely promising for the development of new agricultural tools that could contribute to a more sustainable and secure food production.

## Author Contributions

JB performed the corpus selection and analysis and wrote the first draft of the manuscript. SF and P-LS-C wrote sections of the manuscript. All authors contributed to manuscript revision, read, and approved the submitted version.

### Conflict of Interest Statement

The authors declare that the research was conducted in the absence of any commercial or financial relationships that could be construed as a potential conflict of interest.

## Appendix

**Table:**

**References**	**Culture**	**Training dataset**	**Complexity^**a**^**	**Number of classes**	**Number of images**	**Min–Max number samples/class**
Atabay, 2017	Tomato	PlantVillage subset	^*^	10	19,742	373–5,357
Barbedo, 2018b	12 crop plants	Barbedo, 2016 (15% controlled, 85% in field)	^*^	56	1,383	5–77
Brahimi et al., 2017	Tomato	PlantVillage subset	^*^	9	14,828	325–4,032
Brahimi et al., 2018	14 crop species	PlantVillage	^*^	39	54,323	152–5,507
Cruz et al., 2017	Olive	Own dataset (controlled)	^*^	3	299	99–100
DeChant et al., 2017	Maize	Own dataset (field)	^***^	2	1,796	768–1,028
Ferentinos, 2018	25 crop species	PlantVillage dataset	^*^	58	87,848	43–6,235
Fuentes et al., 2017	Tomato	Own dataset (field)	^***^	10	5,000	338–18,899
Fuentes et al., 2018	Tomato	Own dataset (field)	^***^	12	8,927	338–18,899
Liu B. et al., 2017	Apple	Own dataset (controlled and field)	^*^	4	1,053	2,366–4,147
Mohanty et al., 2016	14 crop species	PlantVillage	^*^	38	54,306	152–5,507
Oppenheim and Shani, 2017	Potato	Own dataset (controlled)	^*^	5	400	265–738
Picon et al., 2018	Wheat	Johannes et al., 2017 extended (field)	^**^	4	8,178	1,116–3,338
Ramcharan et al., 2017	Cassava	Own dataset (field)	^**^	6	2,756	309–415
Sladojevic et al., 2016	Apple, Pear, Cherry, Peach, Grapevine	Own dataset (internet)	NA	15	4,483	108–1,235
Too et al., 2018	14 crop plants	PlantVillage	^*^	38	54,306	152–5,507
Wang et al., 2017	Apple	PlantVillage subset	^*^	4	2,086	145–1,644
Zhang S. et al., 2018	A/ Tomato B/ Cucumber	A/ PlantVillage subset B/ Own dataset (in field)	A/ * B/ **	A/ 8 B/ 5	A/ 15,817 B/ 500	A/ 366 - 5350 B/ 100
Zhang K. et al., 2018	Tomato	PlantVillage subset	^*^	9	5,550	405–814

a *NA, Not Applicable*.

* *Complexity level allowing identification under controlled conditions*.

** *Complexity level allowing identification under uncontrolled conditions*.

*** *Complexity level allowing the development of automatic screening tools*.

**Table:**

**References**	**Classification or detection^**a**^**	**Deep CNN architecture**	**Training strategy^**b**^**	**Best accuracy(%)^**c**^**	**Evaluation quality^**d**^**
Atabay, 2017	C	VGG16, 19, custom architecture	FS–TL	97.53	**
Barbedo, 2018b	C	GoogleNet	TL	87	*
Brahimi et al., 2017	C	AlexNet, GoogleNet	FS–TL	99.18	*
Brahimi et al., 2018	C	AlexNet, DenseNet169, Inception v3, ResNet34, SqueezeNet1-1.1, VGG13	FS -TL	99.76	*
Cruz et al., 2017	C	LeNet	TL	98.60	*
DeChant et al., 2017	D	Custom three stages architecture	FS	96.70	**
Ferentinos, 2018	C	AlexNet, AlexNetOWTBn, GoogleNet, Overfeat, VGG	Unspecified	99.53	*
Fuentes et al., 2017	D	AlexNet, ZFNet, GoogleNet, VGG16, ResNet50, 101, ResNetXt-101	TL	85.98	**
Fuentes et al., 2018	D	Custom architecture with Refinement Filter Bank	TL	96.25	**
Liu B. et al., 2017	C	AlexNet, GoogleNet, ResNet 20, VGG 16 and custom architecture	FS -TL	97.62	*
Mohanty et al., 2016	C	AlexNet, GoogleNet	FS–TL	31	***
Oppenheim and Shani, 2017	C	VGG	Unspecified	96	*
Picon et al., 2018	C	Custom ResNet50, Resnet50	TL	97	***
Ramcharan et al., 2017	C	Inception V3	TL	93	**
Sladojevic et al., 2016	C	CaffeNet	TL	96.3	*
Too et al., 2018	C	Inception V4, VGG 16, ResNet 50, 101 and 152, DenseNet 121	TL	99.75	**
Wang et al., 2017	C	VGG16, 19, Inception-V3, ResNet50	TL	90.40	*
Zhang S. et al., 2018	C	Custom Three Channels CNN, DNN, LeNet-5, GoogleNet	FS	A/ 87.15 B/ 91.16	A/ * B/ *
Zhang K. et al., 2018	C	AlexNet, GoogleNet, ResNet	TL	97.28	*

a *Classification (C)—Detection (D)*.

b *From Scratch (FS)—Transfer Learning (TL)*.

c *If available, the accuracy of the explicitly different test set is privileged*.

d *SSAbsence of three explicit subsets; SSThree explicit subsets; SSSTest set explicitly different from the training set*.
